# Quality of Life Among Veterans With Chronic Spinal Cord Injury and Related Variables

**DOI:** 10.5812/atr.17917

**Published:** 2014-06-14

**Authors:** Mohammad Hosein Ebrahimzadeh, Seyed Hosein Soltani-Moghaddas, Ali Birjandinejad, Farzad Omidi-Kashani, Shahram Bozorgnia

**Affiliations:** 1 Orthopedic Research Center, Mashhad University of Medical Sciences, Mashhad, IR Iran; 2 Department of Orthopedic Surgery, Georgia Regents University, Augusta, USA

**Keywords:** Quality of Life, Spinal Cord Injury, Veteran, Iran

## Abstract

**Background:**

In recent decades, the incidence of spinal cord injuries has increased. In a systemic review on epidemiology of traumatic spinal cord injury in developing countries reported 25.5/million cases per year.

**Objectives:**

To assess the quality of life (QOL) of the veterans among Iran-Iraq war with chronic spinal cord injuries (SCI) and to evaluate long-term impressions of SCI on their quality of life.

**Patients and Methods:**

Fifty-two veterans, all male, with chronic spinal cord injury from Iran-Iraq war (1980-1988) were interviewed and examined. The mean age of veterans at the time of interview was 49.3 years (38 to 80 years). Veterans were assessed by using a 36-item short-form (SF-36), hospital anxiety and depression scale (HADS) and the Barthel index. The presence or absence of pressure sores and spasticity were documented as well.

**Results:**

The mean age of veterans at the time of study was 49.3 years. Pearson's correlation test showed that depression and anxiety have a reverse association with mental component summary (MCS) scale and physical component summary (PCS) scale scores, respectively. Regression analysis showed a negative effect of depression and pressure sore on PCS. Moreover, no association was found between the duration of injury and age with quality of life.

**Conclusions:**

Lower QOL was found among veterans with chronic SCI. More researches on health-related quality of life (HRQOL) are needed to give us a better understanding of changes in life of patients with SCI and the ways to improve them.

## 1. Background

In recent decades, the incidence of spinal cord injuries has increased. In a systemic review on epidemiology of traumatic spinal cord injury in developing countries, Rahimi-Movaghar et al. reported 25.5/million cases per year (95% CI: 21.7–29.4/million/year). According to this report, 82% of all SCIs were male with a mean age of 32.4 years (1). These areas are dealing with many military conflicts every year making spinal cord injuries a major health concern in their communities. SCI is a permanent disability and is associated with decreased life expectancy. Nowadays, with improving life expectancy of patients with SCI, researchers’ main goal is to find a way to improve quality of life (QOL) as well (2). The World Health Organization has described quality of life as “Individuals’ perception of their position in life in the context of culture and value systems in which they live, and in relation to their goals, expectations, standards and concerns”(3, 4). Most experts involved in quality of life studies believe that health-related quality of life (HRQOL) is multidimensional which includes perception of physical, mental, community and role functioning as well as a realization of well-being by individuals (2). In the recent years, many studies were published on different aspects of QOL in patients with SCI, but there are only a few reports from Iran (5-8). The war between Iran and Iraq lasted from September 1980 to August 1988 for around 8 years. This war left about 200000 dead and more than 400000 injured, of whom 2012 had spinal cord injuries (6, 9). In Iran we encounter many patients with SCI secondary to motor vehicle accidents, fall, and veterans from Iran-Iraq war and injured people from the recent wars in the region including Afghanistan and Iraq wars. Regardless of the cause, SCI occurs mostly in young healthy males and associated with permanent disability and decreased level of QOL. Assessment of QOL and problems facing SCI patients would help us identify long-term impressions of SCI and evaluate their needs.

## 2. Objectives

The purpose of this study was to evaluate QOL and related issues in Iranian veterans with chronic war-related SCI living in Mashhad.

## 3. Patients and Methods

A cross-sectional study was performed on Iran-Iraq war veterans with spinal cord injuries in Mashhad, the second most populated city of Iran. Fifty-two (74%) of 72 veterans with chronic spinal cord injury from 1980s war living in the city of Mashhad participated in our survey. All of them were registered in our research center of Mashhad University of Medical Sciences in Mashhad, Iran, and they consented to face-to-face interview. Our inclusion criteria were spinal cord veterans who injured in the battlefields of Iran-Iraq war during 1980 to 1988. Exclusion criteria were veterans with spinal cord injuries who did not agree to participate in the study and veterans who had their spinal cord injury in civil bombardments during the 8-year war. Demographic data of all participants were recorded. Further information regarding educational and recreational activities of the subjects was also collected. Eventually, the study was approved by the Research and Ethical Research Committee of Mashhad University of Medical Sciences. Quality of life (QOL) was assessed using a 36-item short-form health survey (SF-36), a Likert type scale, some with 2 or 3 and others with 5 or 6 points. Moreover, emotional situation of participants and their level of activity were assessed using the hospital anxiety and depression scale (HADS) which is a 4-point Likert type scale and the Barthel index (an ordinal scale), respectively (10). Data related to the duration of injury, the American Spinal Injury Association (ASIA) impairment scale (AIS) (a 5-point ordinal scale) (11), presence or absence of spasticity, pressure sores and colostomy were documented during the visits. SF-36 health survey has 36 items which evaluates eight factors of physical function limitations due to health problems (PF), limitations in social function due to physical or emotional problems (SF), limitations in role activities due to physical health problems (RP), perceived bodily pain (BP), mental health (MH), limitations in usual role activity due to emotional problems (RE), vitality (VT) and general health (GH). Higher scores indicate better health situation. Validity and reliability of Sf-36 have been studied in many countries including Iran (12). Montazeri et al. reported the Iranian version of HADS as a valid and reliable clinical survey in Iran (13).

### 3.1. Statistical Analysis

Chi-square analysis was applied for categorical variables. Fisher’s exact test was applied for 2 × 2 tables to characterize the significance level. When one item was continuous and another one was categorical, analysis of variance (ANOVA) and t-tests were used. A correlation analysis (Pearson's correlation) was performed when both scales were continuous. Kolmogorov-Smirnov test was conducted to examine the normal distribution of data. Descriptive statistics were also used for demographic evaluation. The SF-36 main subscales of our sample were compared to SCI variables using paired t-tests. First, we compared the 8 SF-36 factor scores of veterans with healthy controls. Our second comparison was between the veterans' scores and the presence of spasticity, presence of pressure sores and the Barthel index, anxiety and depression score (HADS), lesion level and the ASIA grades. To find the effect of different domains on the quality of life of participants, regression analysis was used. At first, the ANOVA test was performed to find the applicability of the regression model for PCS and MCS scores. P < 0.05 was considered statistically significant in all analyses.

## 4. Results

All participants were male, married, and injured about 3 decades ago (1980-88). The mean age at the time of SCI was 23.6 years, ranging between 15 and 55 years (SD = 8.2). The mean age of veterans at the time of interview was 49.3 ranging between 38 and 80 years (SD = 7.94). Most of the participants (88.5%) had paraplegia. About one third of paraplegic and tetraplegic veterans had complete spinal cord injury (ASIA A). Regarding the level of education, 4 were higher than high school level, and the rest were at the level of high school or lower. Apart from this, a half of the cases [26 SCI veterans] improved their level of education after occurrence of SCI. In addition to this, more than three-quarters of them [40 SCI veterans] were either retired or unemployed. The remainders were student, teacher or an employee. Sixty-nine percent of the participants were involved in sport activities, and 23% were employed. Regarding factors related to mobility, the mean Barthel index score (activity of daily living) was 57.6 with a range of 0-90 (SD = 23.5). Only two patients were able to walk without assistance. Three veterans used crutches and 47 used wheelchairs for ambulation. Table 1 presents additional demographics.

**Table 1.** Sample Characteristics ^a,b^

Items	Results
**Neurologic classification at discharge from rehabilitation**	
Paraplegia	37 (88.5)
ASIA (A)	13 (32.45)
ASIA (B, C, D)	24 (67.55)
Tetraplegia	6 (11.5)
ASIA (A)	2 (33.3)
**Education status at the time of injury**	
Less than high school	33 (63.4)
High school	15 (28.8)
More than high school	4 (7.8)
**Change in the education status**	
Unchanged	26 (50.0)
High school	14 (27.0)
More than high school	12 (23.0)
**Employment status at the time of interview**	
Unemployed/retired	40 (76.9)
Student	2 (3.8)
Teacher	1 (1.9)
Employee	9 (17.4)
**Mobility**	
Using electric wheelchair	6 (11.5)
Using manual wheelchair	41 (78.9)
Walk with crutches or cane	3 (5.8)
Walk without assistance	2 (3.8)

^a^Abbreviations: ASIA, American Spinal Injury Association.

^b^Data are presented as No. (%).

Associations between independent variables related to SCI and education were examined regarding the two summary scales of the SF-36 (PCS and MCS). The test showed P values of 0.715 and 0.974 for mental component summary (MCS) and physical component summary (PCS) scales, respectively. As a result, we concluded that these scales are normally distributed and can be examined by parametric tests. We found a significant difference in physical functioning as measured by PCS scores between participants with complete and incomplete cord lesions, and those with or without pressure ulcer and/or colostomy (Table 2). Pearson's correlation test was used to examine the correlation of age, time since injury (years), anxiety and depression with PCS and MCS scores (Table 3).

**Table 2.** Effect of Different Variables on Physical Component Summary and Mental Component Summary Scales (One-Sample t-test) ^a^

Items	No. (%)	PCS (Higher, Better)		MCS (Higher, Better)	
		Mean ± SD	P Value	Mean ± SD	P Value
**Neurologic level**			0.482		0.926
Tetraplegia	6 (11.5)	28.3 ± 7.9		50.2 ± 7.0	
Paraplegia	46 (88.5)	30.8 ± 8.2		49.9 ± 11.8	
**ASIA grade**			0.003 ^b^		0.886
Complete (A)	26 (50.0)	27.2 ± 7.4		49.6 ± 11.8	
Incomplete (B, C, D)	26 (50.0)	33.7 ± 7.5		50.1 ± 10.8	
**Educational level**			0.096		0.667
More than high school	15 (28.8)	33.4 ± 9.8		48.8 ± 9.7	
High school or less	37 (71.2)	29.3 ± 7.20		50.3 ± 11.9	
**Income satisfaction**			0.150		0.996
Satisfied	36 (69.2)	31.6 ± 7.0		49.9 ± 11.5	
Non-satisfied	16 (30.8)	28.0 ± 10.1		49.9 ± 11.1	
**Pressure ulcer**			0.007 ^b^		0.587
Yes	13 (25.5)	25.3 ± 5.6		48.41 ± 12.2	
No	39 (75.0)	32.2 ± 8.1		50.4 ± 11.1	
**History of fracture**			0.569		0.248
Yes	21 (40.4)	29.9 ± 7.0		51.4 ± 10.2	
No	31 (59.6)	31.2 ± 8.9		47.7 ± 12.6	
**History of fall**			0.380		0.938
Yes	22 (42.3)	29.6 ± 7.8		49.8 ± 10.8	
No	30 (57.7)	31.6 ± 8.6		50.1 ± 12.2	
**Sport activity**			0.082		0.499
Yes	36 (69.2)	31.8 ± 8.6		49.2 ± 10.9	
No	16 (30.8)	27.5 ± 6.3		51.5 ± 12.3	
**Colostomy**			0.036 ^c^		0.706
Yes	48 (92.3)	22.4 ± 5.6		51.9 ± 18.0	
No	4 (7.7)	31.2 ± 8.0		49.7 ± 10.8	
**Spasticity**			0.146		0.812
Yes	15 (28.8)	31.5 ± 8.5		40.1 ± 10.2	
No	37 (71.0)	27.9 ± 6.7		49.3 ± 13.8	

^a^ Abbreviations: MCS, mental component summary; PCS, physical component summary.

^b^ Statistically significant at the 0.01 level.

^c^ Statistically significant at the 0.05 level.

**Table 3.** Pearson's Correlation Test of Variables ^a^

Variables	PCS	MCS
**Age**		
r ^b^	0.08	0.07
p ^c^	0.562	0.589
**Time since injury, y**		
r ^b^	0.11	0.11
p ^c^	0.435	0.432
**Anxiety**		
r ^b^	-0.05	-0.44 ^d^
p ^c^	0.716	0.001
**Depression**		
r ^b^	-0.37 ^d^	-0.22
p ^c^	0.006	0.113

^a^ Abbreviations: MCS, mental component summary; PCS, physical component summary.

^b^ Pearson’s Correlation of Coefficient.

^c^ Two-tailed P value.

^d^ Correlation is significant at the 0.01 level (2-tailed).

**Figure 1.**
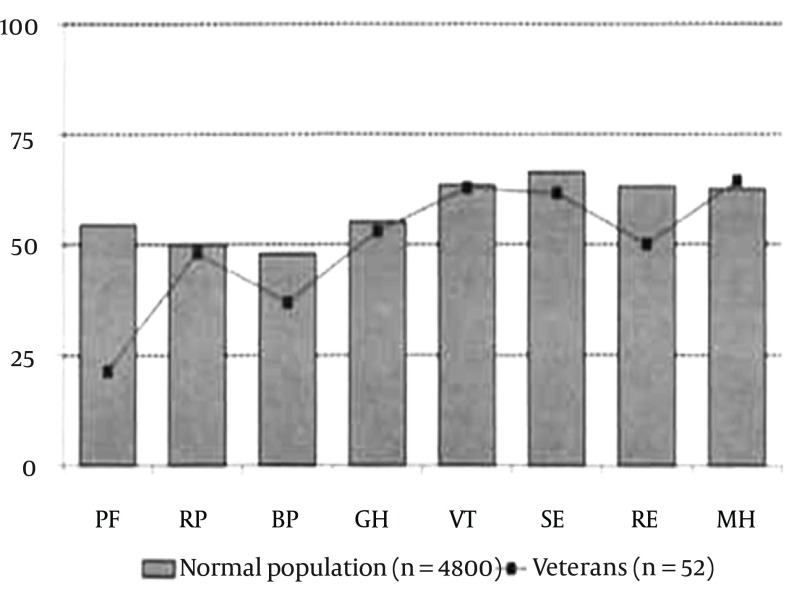
The Short Form (36) Health Survey (SE-36) Score of SCI Veterans Compared to Normal Iranian Population

Higher levels of anxiety were associated with lower PCS scores and depression was negatively associated with MCS scores. We also compared the subscales of the SF-36 between SCI veterans and the normal population (4800 Iranian persons). We concluded a significant difference in scores of these 2 groups except for the vitality index and mental health (Figure 1). To find the associations between different variables on PCS and MCS scales, regression analysis was performed. Regarding PCS, the analysis showed P value of 0.005 which means that one or more variables (anxiety, depression, the Barthel index, neurologic level ([cervical, thoracic and lumbar], age, colostomy, and bed sore) had an impact on the PCS score; however, the test yielded P value of 0.126 for MCS meaning that regression analysis was not applicable to this component. Further analysis revealed that depression and pressure sore had negative effect on the PCS domain (Table 4).

**Table 4.** Regression Analysis for Physical Component Summery

Item	Standardized Coefficient	P Value
**Anxiety**	0.159	0.265
**Depression**	-0.427	0.004
**The Barthel index**	0.056	0.800
**Neurologic level**	0.114	0.602
**Age**	0.067	0.607
**Colostomy**	0.247	0.056
**Bed sore**	-0.307	0.023

## 5. Discussion

There was no difference in PCS score in paraplegic and tetraplegic patients. The same result occurred for MCS. Accessible facilities and recreational programs for tetraplegic patients have helped them overcome structured barriers to social participation. Pearson's correlation test for age, time since injury (year), anxiety and depression showed that anxiety and depression had a reverse effect on MCS and PCS scales, respectively. Similar to studies by Cushman et al. and Barker et al. this test also revealed that there was no association between time since injury and age with quality of life (14, 15). Saadat et al. in the study of HRQOL among veterans concluded that lengthier duration of injury and higher level of education were associated with better PCS and MCS, respectively. On the other hand, they found a negative impression of cervical spinal lesion on MCS and PCS (6). By doing pared t-tests between participants with different levels of education, between those who are satisfied or unsatisfied with their income and between those veterans with or without history of fracture, fall, sport activities, and spasms we could not find a difference in the level of QOL. Having the same level of QOL between participants with different levels of education was also observed in a study by Hu et al. (16). In contrast, the comparison between patients with and without colostomy or bed sore showed that those with either of them have poorer PCS score. The study by Westgren and Levi was consistent with our finding that having pressure sores is associated with lower PF and SF domains (17). Our regression analysis did not find any association between the effects of anxiety or depression, the Barthel index, neurologic level, current age, colostomy, or bed sore on PCS and MCS. No association was found between these variables for MCS (P value = 0.126). However, the P value for the PCS scale in the regression model was 0.005 and further analysis revealed that depression and bedsore both had negative effects on PCS with P values of 0.004 and 0.023 respectively). To find this issue that which components of SF-36 have been affected during a long period since injury (23-31 years), one-sample t-test was performed. It revealed that comparing to the normal population, only the vitality index (VI) and mental health (MH) subscales of veterans had no significant difference with the other groups.

It might be due to supportive community they live in, the support of Foundation of Martyrs and Veterans Affairs, as well as irrelatively high income level. Moreover, from this comparison, it was seen that the pain score of SCI veterans (BP) was almost a half of the normal group. As more perceived pain by veterans can be associated with more anxiety and depression (18) and as a result a lower QOL (19), we predicted lower QOL compared to their normal counterparts. This finding needs more evaluation in future studies using specific scales like the visual analogue scale (VAS). Sixty-nine percent of participants in this study were involved in sport activities, and 23% were employed. Despite the fact, 20% were involved in sports activities and 33% were employed in Tasiemski et al. study (20). In one study by Anneken et al. 51.5% were actively involved in sports. They also concluded that physical activity was the essential impressive factor of QOL, especially within the physical and mental domains (21). In accordance with the results of Saadat et al. the lowest and highest scores were related to PF and MH subscales, respectively (6). Comparison of the main scales of SF-36 between participants with a complete and incomplete injury showed a significant difference in PCS scores (lower scores for veterans with a complete injury). Similarly, 2 different studies in Hong Kong and the USA showed poorer HRQOL in subjects with complete injury (16, 22). All our participants were married. Holicky et al. and Tate et al. showed that married patients with SCI had better mood, higher satisfaction and psychological health, and higher levels of QOL (23, 24). Due to limited number of participants, we were only able to assess a limited number of components of QOL. Further studies are needed to find the effect of marriage in Iranian patients with SCI. Generally, a high rate of marriage (100%) and high rate of satisfaction with income (69.2%) as well as having the vitality index and mental health scores similar to able-bodied people can relatively represent the extent of support provided by the Foundation of Martyrs and Veterans Affairs. These supports have been provided to facilitate their quality of life as well as community reintegration. Future research should include additional aspects of life including community reintegration which plays an important role in our evaluation. By validating the Iranian version of more specific questionnaires like the Craig handicap assessment and reporting technique (CHART) and the spinal cord injury quality of life questionnaire (SCIQL-23), which assess community re-entry and QOL, respectively, we can expand our knowledge regarding the quality of life among SCI people in Iran (8). Due to limited number of participants, the association of the broad-spectrum variables on QOL of patients with SCI could not be assessed comprehensively. As a result, further follow-up studies with larger sample size would give us the opportunity to analyze additional variables. These studies can also assess types of supports from related organizations and possible consequences on quality of life among veterans with SCI.
